# Bidirectional Shifting Effects of the Sound Intensity on the Best Frequency in the Rat Auditory Cortex

**DOI:** 10.1038/srep44493

**Published:** 2017-03-14

**Authors:** Can Tao, Guangwei Zhang, Chang Zhou, Lijuan Wang, Sumei Yan, Yi Zhou, Ying Xiong

**Affiliations:** 1 Department of Neurobiology, Chongqing Key Laboratory of Neurobiology, Third Military Medical University, 30 Gaotanyan St., Chongqing, 400038, China

## Abstract

Frequency and intensity are two independent attributes of sound stimuli. Psychoacoustic studies have found that the sound intensity can affect the perception of frequency; however, the underlying neuronal mechanism remains largely unknown. To investigate if and how the sound level affects the frequency coding for auditory cortical neurons, we recorded the activities of neuronal ensembles and single neurons, as well as the synaptic input evoked by pure tones of different frequency and intensity combinations, in layer 4 of the rat primary auditory cortex. We found that the best frequency (BF) shifted bidirectionally with the increases in intensity. Specifically, the BF of neurons with a low characteristic frequency (CF) shifted lower, whereas the BF of neurons with a higher CF shifted higher. Meanwhile, we found that these shifts in the BF can lead to the expansion of high- and low-frequency areas in the tonotopic map, increasing the evenness of the BF distribution at high intensities. Our results revealed that the frequency tuning can bidirectionally shift with an increase in the sound intensity at both the cellular and population level. This finding is consistent with the perceptual illusions observed in humans and could provide a potential mechanism for this psychoacoustic effect.

Frequency and intensity are two basic aspects of sound information. The frequency determines whether a pure tone sounds “high” or “low”^1^,^2^,^3^, and the accurate perception of frequency allows an animal to process a sound signal, evaluate the degree of risk and then decide whether to run or fight. For humans, frequency is the basis of the appreciation of music and is important for daily speech communication^1^,^4^. The objective evaluation of frequency is the primarily determinant of the perception of pitch, but it is not the only factor involved^4^,^5^. Intensity, which is another attribute of sound that describes its loudness, has been reported to be able to distort the perception of frequency. In 1935, Steven reported that a tone may sound somewhat lower or higher with an increase in intensity, and this is called Steven’s rule^6^. This result suggested that the perception of frequency and intensity are not independent.

Early studies using the Mossbauer technique to measure the vibration of the basilar membrane found that the position of the peak shifted with the sound level^7^, which suggested that the shifting effect originates in the peripheral auditory system. Recent studies observed similar results in cochlear implant recipients^8^, in whom the peak was determined by the position of the stimulus electrodes, suggesting that formation of the frequency shift may not depend merely on the basilar membrane. Moreover, in the ascending auditory pathway, multiple subcortical nuclei participate in the processing of auditory information^9^,^10^,^11^. Whether this biophysical attribute of the basilar membrane is retained in the auditory cortex, as well as how it modulates the responses of auditory cortical neurons remain unclear.

The perception of frequency is dependent on neuronal responses in the auditory cortex^3^,^12^,^13^,^14^,^15^,^16^. Tonal receptive fields (TRFs) represent the fundamental responses to pure tones of various combinations of intensity and frequency^1^. Investigating the TRFs of neurons in the primary auditory cortex (A1) can help to reveal the effect of intensity on frequency perception in the central auditory system. The primary auditory cortex can either faithfully inherit the ascending information from the thalamus^17^ or can enhance or depress specialized functions through local circuits^18^,^19^,^20^,^21^,^22^. Both excitatory and inhibitory synaptic inputs can contribute to the final response of the A1 neurons^23^,^24^. Thus, investigating how thalamocortical synaptic inputs are involved in the interaction between frequency and intensity is of interest.

Here, we observed a significant shift in best frequency (BF) with the increasing intensity for both neuronal ensembles and individual neurons in the primary auditory cortex of both urethane-anesthetized and awake rats. This shift in the BF was also bidirectional. Specifically, the BF of neurons with a characteristic frequency (CF) in a lower frequency range (<32 kHz) shifted left (lower) with the increase in the intensity, whereas the BF of neurons with a CF in a higher frequency range (>32 kHz) shifted right (higher). This systematic bidirectional shift influenced the distribution of the auditory tonotopic map. At high intensity (threshold + 30 dB), the low- and high-frequency areas expanded, which results in higher resolution of frequency at higher intensities. To further investigate the influence of cortical circuits on BF shifts, we performed *in vivo* whole-cell patch-clamp recordings in layer 4 (L4) neurons in A1. We found that the BF shifts were observed in both excitatory and inhibitory synaptic inputs, implying that the BF shift is inherited from the thalamus and retained in the cortex. Together, our results demonstrate the influence of sound pressure on the frequency at the single neuron and population level in the primary auditory cortex.

## Results

### Bidirectional Best Frequency Shift of Neuronal Ensembles

**Figure 1:**
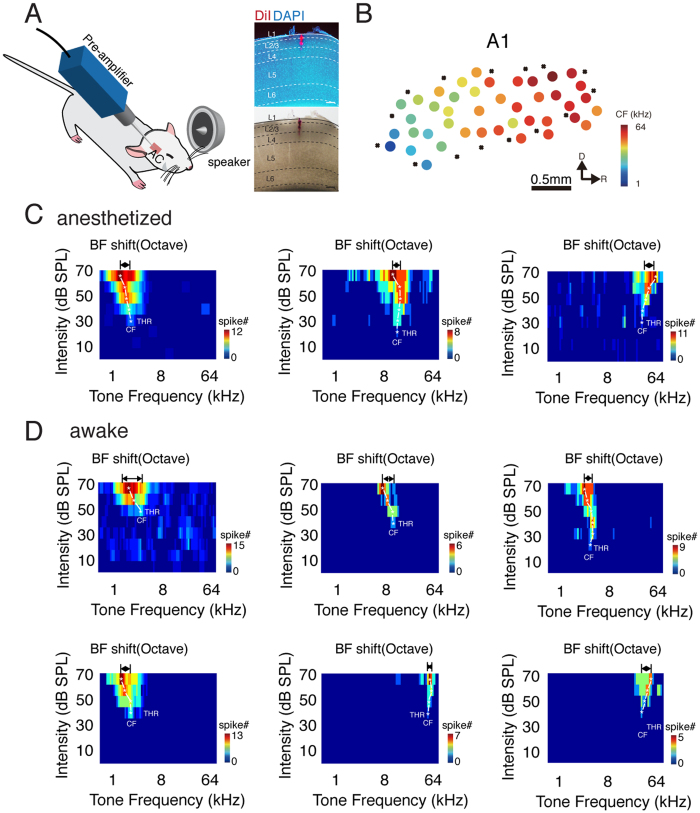
BF of the Multiunit Response Shifts with Intensity. (**A**) Schematic of the recording setup. Right panel, track of the recording tungsten electrode. Scale bar, 200 μm. (**B**) An example of a tonotopic map of the primary auditory cortex. Each dot represents a recording site. The color represents the CF. A cross indicates no response to white noise at 70 dB SPL. D, dorsal; R, rostral. A1, primary auditory cortex. (**C**) Example of the TRFs with a low (left), middle (middle), and high (right) CF. The color represents the number of spikes. The white stars indicate the BF at each intensity level. THR, intensity threshold. The horizontal arrows indicate the difference between the BF at 70 dB SPL and the BF at the threshold level. (**D**) Example TRFs obtained in awake rats with their heads in a fixed position.

Previous behavioral studies have reported that frequency perception is not consistent when the sound intensity changes^25^,^26^,^27^. Sound perception is based on neuronal activity in the auditory cortex^1^. Therefore, we asked whether the frequency preferences of auditory cortical neurons would change with the stimulus intensity. We first conducted *in vivo* multiunit extracellular recordings between 420 μm to 450 μm, which corresponds to L4 in A1, in anesthetized rats (Fig. 1A, see the Methods for details) to obtain the TRFs of neuronal ensembles. We used a micromanipulator to monitor the position of the electrode and we further confirmed the depth after some experiments (Fig. 1A). The TRF is composed of the responses evoked by 568 short pure tones (0.5–64 kHz, 0.1-octave step; 0–70 dB, 10 dB step, 35 ms duration with 5 ms cosine ramp). The BF represents the frequency that evokes the highest firing rate of the neuronal ensembles^28^. The CF equals the BF at the threshold intensity level. The frequency preferences of the entire A1 region can be mapped based on the CF of each recording site, producing what is known as a tonotopic map (Fig. 1B). At each recording site, the evoked responses can be detected as the average activity of the local neuronal ensembles. Fig. 1C shows three examples of recorded responses at sites with low, medium and high CFs. We can see that the BF is not consistent and shifts either leftward or rightward with the increasing intensity. We also observed a similar shift pattern in the A1 region of awake rats (Fig. 1D).

**Figure 2:**
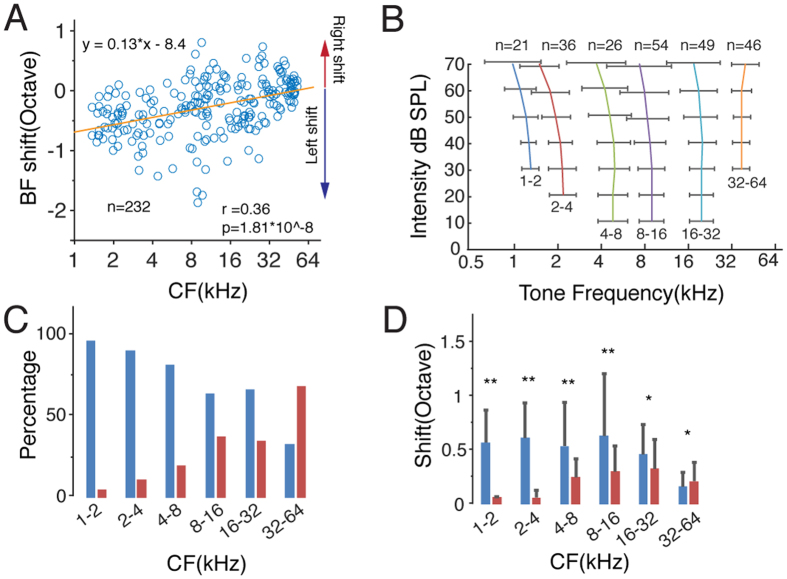
The Best Frequency Shift Depends on the Characteristic Frequency. (**A**) Correlation between the CF and the BF shift. The orange line is the linear curve fit. A positive value for the BF shift represents a shift to a higher frequency, whereas a negative value represents a shift to a lower frequency. R, Pearson correlation coefficient. (**B**) BF shift curves for the groups of different CFs. Bar, S.D. (**C**) Percentage of shift direction for each group. Blue, shift to a lower frequency; red, shift to a higher frequency. (**D**) Range of shift for each group. Blue, shift to a lower frequency; red, shift to a higher frequency. Bar, S.D. **p < 0.01, *p < 0.05, t-test.

According to previous behavioral studies, increases in the sound intensity level lead high-frequency sounds to seem higher and low-frequency sounds to seem lower^2^,^6^. Thus, we probed the relationship between the direction of the BF shift and the CF. We compared the BF at threshold and the BF at the 70 dB level at each recording site and used the frequency difference between these two intensity levels (BF difference) to evaluate the direction and degree of the shift (Fig. 1C,D). A positive value indicates that the BF shifted rightward, that is, to a higher frequency. Our results show there is a positive correlation between the BF shift of a neuronal ensemble and its CF (Pearson correlation coefficient, r = 0.36, p = 1.81 * 10^−8^, Fig. 2A). The neuronal ensembles with relatively high CFs (>32 kHz) shifted to higher frequencies, whereas those with relatively low CFs shifted to lower frequencies. It should be noted that the frequency for sound stimulation ranges from 0.5 kHz to 64 kHz, which could lead to an underestimation of the shifting effect for the high-frequency group. To further clarify the relationship between the CF and the BF shift, we grouped the neuronal ensembles according to their CFs (Fig. 2B). When the sound intensity increased, the groups with CFs lower than 32 kHz generally shifted to lower frequencies, whereas the groups with CFs higher than 32 kHz shifted to higher frequencies. Next, evaluating the proportions of the shift directions within each group (Fig. 2C), we found that both right-shifted and left-shifted neuronal ensembles were presented in each group but that they occurred at different ratios. As the CF increased, the percentage of left-shifted neuronal ensembles decreased, whereas the percentage of right-shifted neuronal ensembles increased. Additionally, left shifting was dominant for the groups with CFs lower than 32 kHz, but that right shifting was dominant in the groups with CFs higher than 32 kHz. We also measured the range of the shifts within each group (Fig. 2D). We divided the multiunit responses into left-shift and right-shift clusters for each group and then performed Student’s t-test within each group to compare the range of shifting effect. The range of leftward shifting was generally larger than that of rightward shifting in the low-to-middle frequency range. Together, these results indicate that the frequency preference of the neuronal ensembles is not independent of the sound intensity level; instead, as sound intensity increases, the low CF neuronal ensembles tend to prefer even lower frequencies, and the high CF neuronal ensembles tend to prefer higher frequencies.

### Cortical Areas Representing Low and High Frequencies Extend with Increasing Intensity

**Figure 3:**
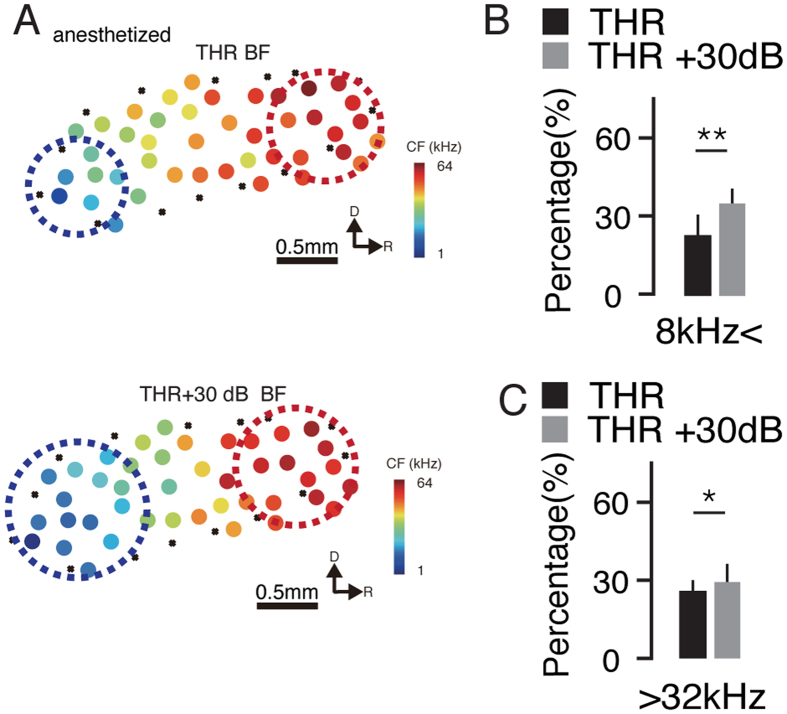
Tonotopic Maps Change with the Intensity. (**A**) Tonotopic map at threshold intensity (upper) and at 30 dB above threshold (lower). The dotted circles indicate the low (blue) and high (red) frequency area, respectively. (**B**) The area ratio of the low frequencies (percentage of recording sites with a BF below 8 kHz in all the recorded sites) at threshold intensity and at 30 dB above threshold. **p = 0.0087, paired t-test, n = 16. (**C**) The area ratio of the high frequencies (percentage of recording sites with a BF above 32 kHz in all the recorded sites) at threshold intensity and at 30 dB above threshold. *p = 0.037, paired t-test, n = 16.

Tonotopic maps are correlated with sound processing and perception, and alterations in tonotopic maps correlate with discrimination behavior^20^,^29^, neuronal plasticity^30^ and development^31^. Tonotopic maps are composed of the CFs of multiple sampling sites in the auditory cortex (Fig. 1B) and therefore represent the general frequency preferences of the entire A1 region at the threshold intensity level. Because the dependence of the frequency perception on the sound intensity likely affects the tonotopic map, we used the BF at 30 dB above threshold, rather than the CF, to reconstruct the tonotopic map and then compared that map with the one based on the CF (Fig. 3A). We calculated the area preferring low frequency (BF < 8 kHz) at the threshold intensity (quantified as the proportion of the total recording sites that showed a BF lower than 8 kHz) and compared it with the area obtained at 30 dB above threshold (Fig. 3B). We found that the fraction of the map devoted to the low-frequency sound increased from 22.3 ± 7.4% (mean ± SD unless otherwise specified) at threshold to 35.3 ± 5.4% at 30 dB above threshold (p < 0.01, paired t-test). This same calculation was then applied to the area preferring high-frequency sound (Fig. 3C, BF > 32 kHz). Consistent with the low-frequency area, the fraction of the map devoted to high-frequency sound increased from 26.3 ± 2.6% to 29.1 ± 6.3% (p < 0.05, paired t-test) at 30 dB above threshold. Thus, the areas preferring low- and high-frequencies expanded with the increasing sound intensity.

### Frequency Preference of Pyramidal Cells Shifts with Increasing Intensity

**Figure 4:**
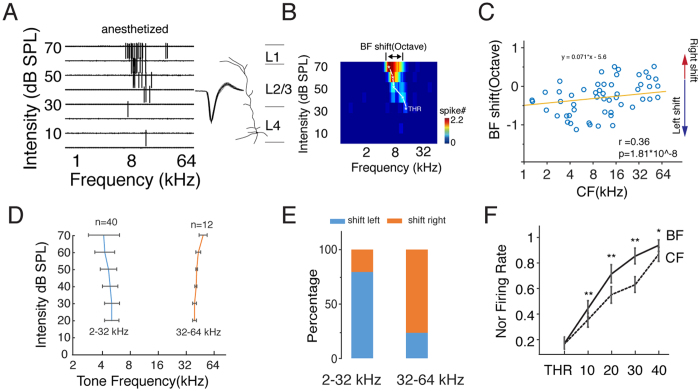
BF of Single Pyramidal Neurons Shifts with the Intensity. (**A**) TRF of a single excitatory neuron. Left, spike tonal receptive field composed of 568 traces in response to tones at different frequencies and intensities. Right panel, 50 randomly chosen superimposed spike waveforms and the reconstructed morphology of a L4 pyramidal neuron. (**B**) Color map of the TRF. The color represents the averaged spike number. The white stars represent the BFs at the different intensities. The horizontal arrow indicates the difference between the BF at 70 dB SPL and at threshold intensity. (**C**) Raster plot of the CF versus the BF shift for each recorded neuron, n = 52. (**D**) Averaged BF shift curve for the low- and high-CF groups. Bar, S.D. (**E**) Percentage of shift directions for the low (2–32 kHz)- and high (32–64 kHz)-CF groups. (**F**) Normalized firing rate at the BF (solid line) and CF (dashed line) at each intensity (threshold to 40 dB above threshold), **p < 0.01, *p < 0.05, paired t-test.

**Figure 5:**
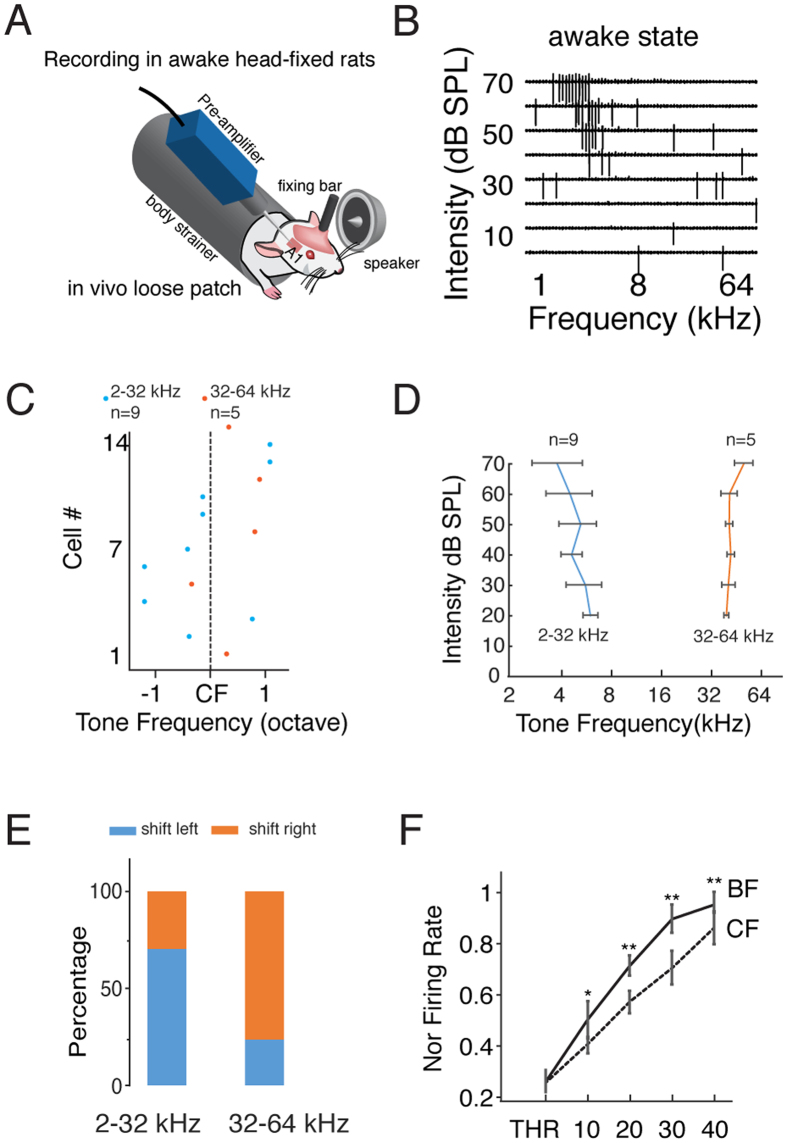
BF of Single Pyramidal Neurons Shifts with Intensity in Awake Animals. (**A**) Schematic drawing of a loose-patch recording in an awake rat. (**B**) An example spiking tonal receptive field of a regular-spiking neuron in A1. (**C**) Raster plot of the BF shift for each recorded neuron. The blue dots represent the neurons with a CF between 2 and 32 kHz (n = 9), and the orange dots represent the neurons with a CF between 32 and 64 kHz (n = 5). The CF of each neuron has been aligned (dashed line). (**D**) Averaged BF shift curve for the low- and high-CF groups. Bar, S.D. (**E**) Percentage of shift directions for the low (2–32 kHz)- and high (32–64 kHz)-CF groups. (**F**) Normalized firing rate at the BF (solid line) and CF (dashed line) at each intensity (threshold to 40 dB above threshold), **p < 0.01, *p < 0.05, paired t-test.

Multiunit responses represent the averaged activity of the neuronal ensembles^32^. However, the activity of single neurons can differ from that of the neuronal ensemble^33^. Therefore, we performed *in vivo* cell-attached loose-patch recordings to obtain single neuronal responses to tone stimulation and to detect the BF shifts with the intensity changes (see the Methods for details). Figure 4A shows an example of single-cell spiking responses evoked by 568 pure tones. We identified pyramidal neurons based on the trough-to-peak interval (0.54 ± 0.12 ms) and morphological reconstructions (Fig. 4A, see the Methods for details). We found that the BFs of the single neuronal responses also changed with increased intensity (Fig. 4B). Based on the results of the multiunit responses (Fig. 2B,C), we classified the recorded neurons into two groups. We plotted the shift range of the BF at 70 dB versus the BF at threshold level (Fig. 4C). The neurons with CFs lower and higher than 32 kHz are shown in blue and orange, respectively. Similar to the multiunit responses, most of the neurons with CFs lower than 32 kHz tended to shift to lower frequencies, whereas those with CFs higher than 32 kHz tended to shift to higher frequencies (Fig. 4C). Thus, we plotted the averaged BF shift curves for the two groups (Fig. 4D). We also calculated the percentages of left- and right-shifted neurons in each group and found that of the neurons with a CF below 32 kHz, 78% shifted to a lower frequency, and 22% shifted to a higher frequency, whereas among the neurons with CF above 32 kHz, 25% shifted to a lower frequency and 75% shifted to a higher frequency. These results showed a dramatic difference in the percentages of neurons whose BF shifted leftward, contributing to the BF shifts of the neuronal ensembles (Fig. 2C). At the threshold intensity level, the BF is equal to the CF, but the BF does not overlap with the CF at higher intensities. Because the BF is determined by the firing rate evoked by sound stimulation, a shift in the BF indicates a shift in the frequency eliciting the highest firing rate. Therefore, we calculated the firing rate at intensities above threshold. We found that the firing rate monotonically increased for both the CF and BF; however, the rate of increase was higher for the BF than for the CF, contributing to the difference between the firing rates at the BF and the CF at higher intensity levels (Fig. 4F). Similar results were observed in awake animals, which suggested that this bidirectional shifting effect was not an artifact caused by anesthesia (Fig. 5).

### BF Shifts Change the Distribution of Frequency Preference at High Intensities

**Figure 6:**
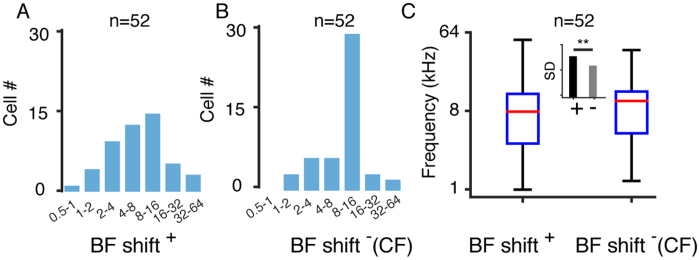
BF Shift Expands the Hearing Range. (**A**) BF distribution at 70 dB SPL. (**B**) BF distribution without shift (equal to the CF) at 70 dB. (**C**) Boxplot of the BF and CF distributions for single neurons. Inset, SD of BF and CF distribution, **p < 0.01, F-test.

Increases in the sound level lead to the activation of more neurons in the low- and high-frequency ranges. Next, we plotted the distribution of the BFs of the recorded single neurons at 70 dB (Fig. 6A). If the BF does not shift with the sound level, then the BF at 70 dB will be equal to the CF. We then plotted the distribution of the BF without the shift (CF) at 70 dB (Fig. 6B). The standard deviation of the distribution of the BFs with the shift was significantly larger than that without the shift (Fig. 6C p < 0.01, K-S test to test the equality of variation). We found that the BF with the shift at 70 dB was more evenly distributed than that without the shift (Fig. 6A,B). These results suggested that the BF shift smooths the distribution of the BFs at higher sound intensities and increases the efficiency of the frequency perception.

### Shifted Excitation and Inhibition Ratios Contribute to the BF Shift

**Figure 7:**
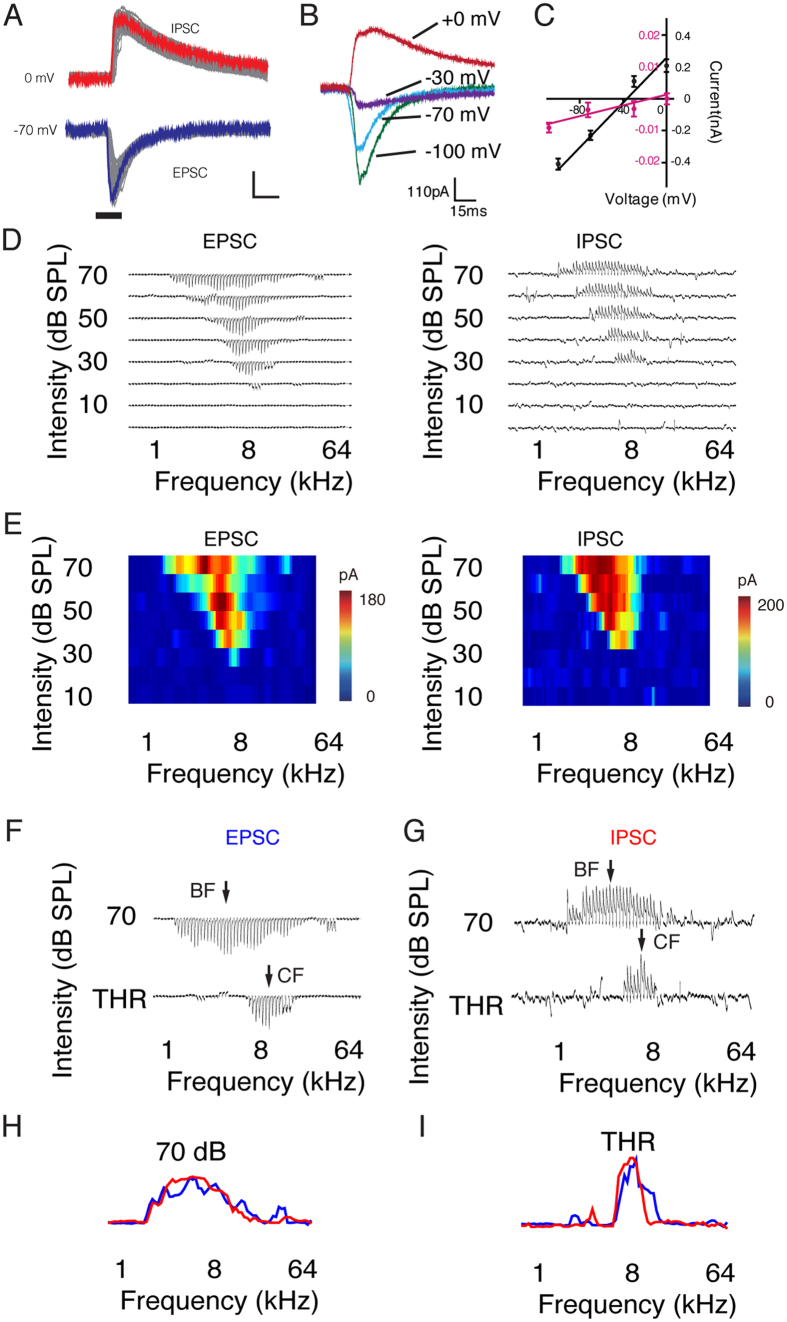
Excitatory and Inhibitory Synaptic Inputs Shift Equally. (**A**) Example of sound-induced excitatory and inhibitory synaptic inputs. The bars represent the sound stimulation. Scale bar, 50 ms and 120 pA; EPSC, excitatory postsynaptic current; IPSC, inhibitory postsynaptic current. (**B**) Synaptic responses evoked by 60 dB white noise at different holding potentials. (**C**) I-V curves for the synaptic currents. These curves were based on the synaptic amplitude within 0–1 ms (red) and 20–21 ms (black) windows after the synaptic onset. Error bar, SEM. (**D**) Example synaptic tonal receptive field of the EPSCs and IPSCs. (**E**) Color map of the synaptic tonal receptive field. The color represents the amplitude of the evoked currents. (**F** & **G**) Enlarged synaptic input tuning curve of the EPSCs and IPSCs at threshold and 70 dB. The arrows indicate the BF and CF. (**H** & **I**) Enveloped tuning curves for the EPSCs (blue) and IPSCs (red) at threshold and 70 dB.

Neurons in the auditory cortex receive both excitatory and inhibitory synaptic inputs^23^,^24^, which have been reported to alter the tuning profiles of excitation and inhibition^21^,^34^. To investigate the synaptic mechanisms underlying the BF shifts in the auditory cortical neurons, we performed *in vivo* whole-cell voltage-clamp recordings to dissect the excitatory and inhibitory synaptic inputs in the thalamorecipient L4 (see the Methods for details). The membrane potential of the recorded neurons was clamped at −70 mV and 0 mV (Fig. 7A), which are the reversal potentials for inhibitory and excitatory ions, respectively (Fig. 7B,C)^17^. We then obtained the excitatory and inhibitory postsynaptic currents evoked by tones of different frequencies and intensities (Fig. 7D). We found that the BFs of both the excitatory and inhibitory synaptic inputs shifted at higher intensity (Fig. 7D–G). However, the tuning profiles for excitation and inhibition were largely matched (Fig. 7H,I).

**Figure 8:**
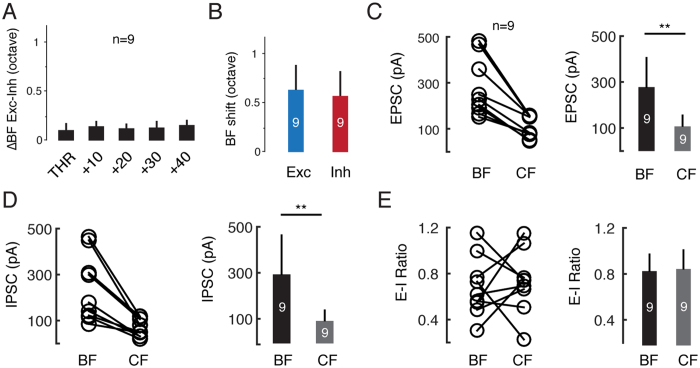
Synaptic Input Determines the BF Shift. (**A**) Difference in the BF between excitation and inhibition at each intensity. No significant difference was observed. (**B**) The BF shift at 70 dB for both excitation and inhibition. Error bar, SD. (**C**) Amplitude of the EPSCs at the BF and CF at 70 dB. Right panel, average and SD, **p < 0.01, paired t-test. (**D**) Amplitude of the IPSCs for the BF and CF at 70 dB. Right panel, average and SD, **p < 0.01, paired t-test. (**E**) Excitation and inhibition amplitude ratio of the BFs and CFs at 70 dB. Right panel, average and SD, **p < 0.01, paired t-test.

We next compared the difference in the BF between excitation and inhibition at intensities above threshold; however, no significant differences were observed (Fig. 8A). In addition, no significant difference was found between the BF shifts (absolute difference between the BF at 70 dB and at threshold) for excitation and inhibition (Fig. 8B). Next, we compared the synaptic currents for the BF and CF at 70 dB and found that both the excitatory and inhibitory synaptic currents at the BF were significantly larger than those at the CF (Fig. 8C,D). However, at 70 dB, no significant difference was observed between the ratio of excitatory and inhibitory synaptic currents for the BF and CF (Fig. 8E). These results demonstrate that with increasing intensity, the BF shifts simultaneously for both excitatory and inhibitory synaptic inputs.

### BF of Local Interneurons Shifts with Intensity

**Figure 9:**
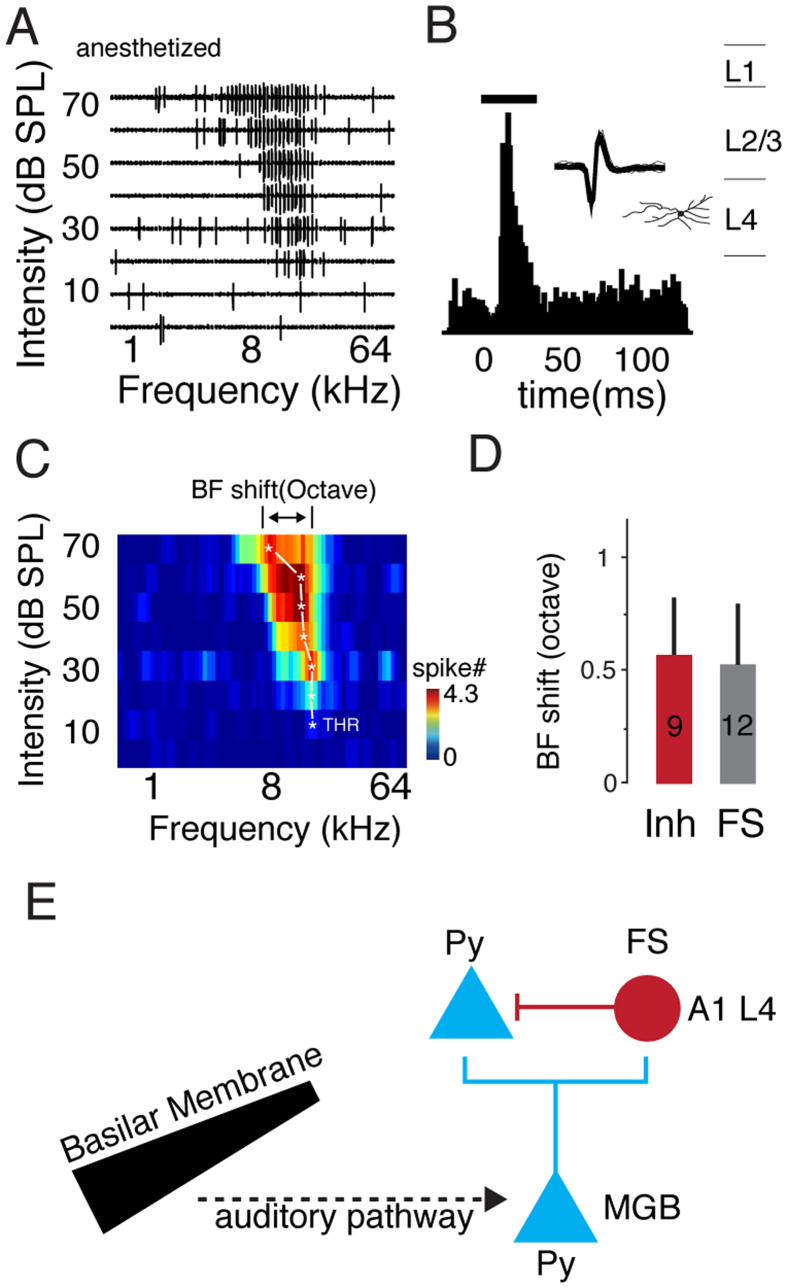
BF Shift of Fast Spiking Neurons. (**A**) Tonal receptive field of single fast spiking neurons. Left, spike tonal receptive field composed of 568 traces in response to tones at different frequencies and intensities. (**B**) Peristimulus time histogram. The bar represents the sound stimulation. Right corner, 50 randomly chosen superimposed spike waveforms and the reconstructed morphology of a L4 fast-spiking neuron. (**C**) Color map of the TRF. The color represents the number of spikes. The white stars represent the BF at each intensity. The horizontal arrow indicates the difference between the BF at 70 dB SPL and the intensity threshold level. (**D**) Difference between the BF at the threshold level and 70 dB SPL. Inh, inhibitory synaptic input; FS, fast spiking neurons. Bar, S.D. (**E**) Schematic model representing the co-shifting of the excitatory and inhibitory synaptic inputs, which supports the feedforward inhibitory synaptic circuit in L4 of A1 and suggests that the BF’s dependence on the intensity is inherited from the early stages in auditory processing, including the basal membrane response properties.

Inhibitory synaptic inputs into L4 pyramidal neurons largely originate from local inhibitory interneurons^17^,^24^,^35^. The BF shift of the inhibitory synaptic inputs suggested that the BF of the local inhibitory interneurons should also shift with changing intensity. Thus, we used *in vivo* loose-patch recordings in the auditory cortex to record the spiking TRFs of interneurons (Fig. 9A,B). The cell types were identified based on the trough-to-peak intervals (0.31 ± 0.08 ms) and morphological reconstruction after the recordings in several experiments, as previously reported^24^,^35^ (see Methods for details). As expected, we found that the BF of the interneurons also shifted with the intensity (Fig. 9C), and no significant differences were observed between the range of the spiking BF shift (difference in BF between threshold and 70 dB) for the interneurons and the BF shift of the inhibitory synaptic inputs to the pyramidal neurons (Fig. 9D).

## Discussion

The preferred frequency of neuronal ensembles shifted downwards (lower) or upwards (higher) with an increase in the sound intensity, which led to a systematic change in the auditory tonotopic map with the increasing sound pressure. The single neuronal responses showed that the BF shifted either higher or lower. However, the percentage of left-shifting neurons was much higher in the low CF area, whereas the percentage of right-shifting neurons was much higher in the high CF area, contributing to the BF shift for the neuronal ensembles. Moreover, we found that the BF of the synaptic inputs also shifted with an increase in the sound intensity and that the excitatory and inhibitory synaptic inputs were co-tuned. We also recorded the spiking responses of single interneurons in L4 of the A1, which provides much of the feedforward inhibitory synaptic input for L4 pyramidal neurons^17^,^21^; these cells also showed the BF shift. These results suggested that the BF shift is inherited from the ascending auditory pathway, retained in the auditory cortex and able to reconstruct the tonotopic map at different levels of sound intensity.

The influence of the sound intensity on pitch perception was first studied in humans almost a century ago. Steven reported in 1935 that with an increase in the intensity, high-frequency tones sound higher, and low-frequency tones sound lower^6^. Psychoacoustic studies also reported significant variation among individuals^36^,^37^, which leads to a discrepancy in the degree to which the intensity influences the pitch^38^,^39^,^40^. However, in the naïve rat auditory cortex, we found the BF shift to be generally consistent. This difference might be due to the high plasticity of auditory cortical neurons^41^,^42^,^43^. Based on previous experiments^2^, Moore proposed that the neuronal system might be able to compensate for changes in pitch and thus that the sense of pitch for humans living in an enriched sound environment is likely very diverse.

Our results showed that the cortical areas representing the high and low frequencies expanded with the increasing sound pressure. Previous studies have suggested that the tonotopic map is highly correlated with the sound discrimination ability and that pairing sounds of a certain frequency with a reward or punishment will dramatically increase the size of the area representing that frequency^44^,^45^. The change in the structure of the tonotopic map might therefore give rise to the psychoacoustic frequency illusion. Interestingly, our results showed that this shifting effect also increased the ability to process high- and low-frequency sounds at higher intensity levels. Meanwhile, the intensity threshold is higher for high and low frequencies than for middle frequencies, which is an attribute of the middle ear^4^,^46^. Thus, a shift in the preferred frequency leads to higher frequency resolution at higher intensities. This effect may be beneficial, and this systematic change is therefore transmitted through several stations of the auditory pathway and maintained in the auditory cortex.

*In vivo* loose-patch recordings allowed us to obtain responses from single neurons, and the shift directions were not completely homogeneous. We still found right-shifting neurons in low-frequency areas and left-shifting neurons in high-frequency areas. However, the proportions of these two groups of neurons reversed when the CF of the neurons changed from low frequency to high frequency, which determined the shifting direction at the population level. Although we do not know which factors generate this heterogeneous shift pattern, it may be generated in processing through the auditory pathway. Therefore, the position of the peak response in the basilar membrane cannot fully explain the BF shift.

The spiking activity of pyramidal neurons arises from the integration of excitatory and inhibitory synaptic inputs^47^. Previous studies have shown that excitation and inhibition can alter the tuning profiles of cortical neurons^22^,^23^,^24^,^48^,^49^. Our results showed that excitatory and inhibitory synaptic inputs are co-tuned, and both components show similar levels of BF shift with the increasing intensity. Due to space-clamp issue, the amplitude of excitatory and inhibitory synaptic input might be underestimated^23^. However, this limitation would not change the main finding of this paper, because this effect would equally attenuate the response evoked by each tone stimulation, thus would not change the BFs of these recorded neurons. Previous studies have shown that L4 neurons mainly receive excitatory synaptic inputs from the ventral part of the medial geniculate body^1^ and local interneurons, which also receive inputs from the thalamus to provide feedforward inhibition for L4 pyramidal neurons^24^. The shift pattern of the auditory cortical neurons is generally inherited from the thalamus and might be generated from the inner ear (Fig. 9E). Here, we used pure tones with different frequency and intensity combinations to investigate if and how the intensity affects the frequency tuning. However, pitch is a property that is not exclusive for pure tones. In the environment, vocal sound is more spectrally complicated and consists of a series of harmonics. Previous studies have suggested that the pitch of complex sounds is based on the lowest harmonic of complex sound^4^. How the intensity of complex sounds affects pitch perception needs further investigation.

To probe the function of the BF shift, we analyzed the BF distribution at high intensities with and without shifts (equal to CF). We found that the shift effect increased the evenness of the BF distribution. The BF is the frequency that evokes the most robust spiking response and the shortest onset latency^22^,^24^. The increased evenness of the BF distribution allows for more high- and low-frequency sounds to be within the BF range, thereby potentially increasing the effective frequency-coding range. Studies of the middle ear have found that the middle transfer function is band limited and centered at the middle frequencies, thus elevating the threshold for high and low frequencies^4^,^5^,^46^. Shifting the BF as the intensity changes might be a strategy by which the auditory system can partially compensate for the lost bandwidth in the high- and low-frequency ranges. Previous studies observed a similar pitch illusion in cochlear implant recipients and hypothesized that this shift in pitch must be considered in future speech-coding strategies^8^. However, our results suggest that this type of pitch shift might be beneficial for expanding the audibility of the frequency range.

## Methods

### Animals and Surgical Procedures

All procedures were approved by the Animal Care and Use Committee of the Third Military Medical University. All experiments were performed in accordance with the relevant guidelines and regulations. Sprague-Dawley rats (female, 2 months old, 160–200 g) were used in this study. The animals were first anesthetized using urethane (1.5 g/kg), with an additional dose administered if hind paw retraction was evoked by toe pinch. The animal’s body temperature was continuously monitored and maintained at 37 °C using a heating pad with a feedback controller. The animals were head-fixed using a customized apparatus. A standard craniotomy procedure was used to expose the right auditory cortex (bregma −2 mm to −7 mm on the temporal skull). The recordings were performed in awake animals according to previously described methods^24^. Briefly, the animals were anesthetized using isoflurane (2%), and a metal fixing bar was then attached to the skull using dental cement. After the craniotomy, the window was covered using Kwik-Cast Sealant (WPI Inc., USA). The animals were then returned to their cages. The recordings were performed after the animals were habituated to the recording stage for more than 30 min.

### Sound Calibration and Generation

A customized LabVIEW (2014 edition, National Instruments, USA) program was used for the signal waveform generation and sound calibration. The auditory stimuli were delivered via a free-field magnetic speaker (MF1, TDT Inc., USA), which was driven by a stereo power amplifier (SA1, TDT Inc., USA). A 1/4″ pressure microphone and a prepolarized condenser (377A01 microphone + 426B03 preamplifier +480E09 signal conditioner, Piezotronics Inc., USA) were used for the sound calibration. A high-speed DAQ board (1 MHz/s sampling rate, PCI-6251, National Instruments, USA) was used to record the signals. To obtain the TRF, 568 pure tones (0–70 dB SPL, 10-dB step; 0.5–64 kHz, 0.1 octave step; 35 ms duration; 5 ms sine ramp; 250 ms inter-stimulus interval) were pseudo-randomly delivered and counted as one trial.

### *In Vivo* Electrophysiology

#### Multiunit Extracellular Recordings

Multiunit spike responses were obtained using Parylene-coated tungsten electrodes (100 kΩ, WPI Inc., USA), and the signals were amplified and recorded on a TDT System (gain: 20000, sampling rate: 50 kHz, TDT Inc., USA) and band-pass filtered (300/3000 Hz, high pass/low pass). The spikes were detected automatically using Brainware (TDT Inc., USA). The electrode position was controlled and monitored using a micromanipulator (DMA-1511, Narishige, Japan). In some experiments, we coated the tungsten electrode with DiI (ThermoFisher, USA) and checked the position of the electrode after the recording (Fig. 1A). A1 was functionally identified based on the tonotopic gradient^17^,^24^. The experiments were conducted in a double-shielded sound booth (Shenyang Sound-Proof Booth Factory, China).

#### Patch-Clamp Recordings

Single neuronal spike responses were obtained using loose-patch recordings based on previously described methods^24^. Briefly, a glass pipette (impedance: 4–7 MΩ, Sutter, Inc., USA) filled with artificial cerebrospinal fluid (ACSF, in mM: 124 NaCl, 3 KCl, 2.6 NaHCO, 2 CaCl, 2 MgCl, 1.23 NaH_2_PO_4_, 10 glucose, and 2% biocytin, pH 7.25) was used. Positive pressure (4 psi) was used to penetrate the pia matter, after which the pressure was reduced to 0.4 psi. An increase in the pipette impedance and spiny baseline indicated that the tip of the pipette was approaching a neuron. The pressure was then released to form a loose seal (20–150 MΩ). Agarose was applied to maintain moisture and minimize cortical pulsation. The single neuronal spike signals were recorded using an amplifier (EPC −10 HEKA Gmbh, Germany) under voltage-clamp mode. Excitatory pyramidal neurons and inhibitory interneurons were identified by the trough-to-peak interval of the spike waveform and further identified by biocytin-based histological reconstruction after several recordings^24^.

The synaptic input responses were acquired via *in vivo* whole-cell patch-clamp recordings. The experimental procedure was similar to that for the loose-patch recordings. The internal solution contained (in mM) 125 Cs-gluconate, 5 TEA-Cl, 4 MgATP, 0.3 GTP, 10 phosphocreatine, 10 HEPES, 1 EGTA, 2 CsCl, 1.5 QX-314, and 1% biocytin (pH 7.2). The fast and slow capacitances were completely compensated, and an effective series resistance between 10 and 25 MΩ was achieved by compensating for 45–50% of the series resistance (20–50 MΩ). The signals were sampled at 10 kHz (EPC 10, HEKA Gmbh, Germany). Command voltages of −70 mV and 10 mV were given for the excitatory and inhibitory currents, respectively. The data with a neuronal resting membrane potential more positive than −55 mV or an unstable series resistance (>20% change during the course of the recording session) were excluded from further analysis. As reported and discussed previously^21^,^24^, our whole-cell recordings exclusively targeted excitatory pyramidal neurons; this specific targeting was further confirmed by morphological reconstruction of neurons after the recordings. Previous studies suggested that space clamping and the cable effect could underestimate synaptic conductance, but this would not affect our results because we mainly compared the relative tuning properties of the excitatory and inhibitory synaptic inputs.

### Histology

After the loose-patch recording experiments, a current pulse train was applied (1 nA, 200 ms on, 200 ms off; duration, 30 min). The rat was then deeply anesthetized with urethane (i.p.) and perfused transcardially with saline and 4% paraformaldehyde. After decapitation, the brain was kept in 4% paraformaldehyde for at least 24 h at 4 °C. Then, the brain was sectioned into 200-μm thick slices using a vibratome. The sections were rinsed in PBS, and peroxidase activity was then blocked using hydrogen peroxide (1%). The sections were then incubated with an avidin–biotin HRP complex (ABC Kit, Vector Laboratories, Inc., USA) at 37 °C for 2 h. After the sections were thoroughly rinsed in PBS, they were transferred into a DAB solution (Vector^®^ SG Substrate Kit, Vector Laboratories, Inc., USA), and the immunoreaction was monitored using a microscope. After reconstruction, the recorded neurons were classified based on the pyramidal-shaped somas and apical dendrites. For the whole-cell recordings, no electrical pulse train was applied. For the experiments to determine the position of the recording electrode, the brain was sectioned as described above. Then, the brain slices were rinsed in DAPI (1:1000 in PBS, ThermoFisher, USA) for 5 min. The images were obtained using a fluorescent microscope (Olympus, Japan). The layer of the cortex was determined using the rat brain atlas^50^.

### Data Analyses

The online analyses were performed using commercial software: Brainware (TDT Inc., USA) for the extracellular recordings and Patch Master (HEKA Gmbh, Germany) for the *in vivo* patch-clamp recordings. The offline analyses were performed using customized Matlab scripts (MathWorks, USA). SPSS (IBM, USA) was used for the statistical analyses. The excitatory and inhibitory synaptic conductances were derived according to previous reports^21^,^24^,^35^ using the following equation:


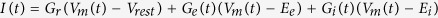


where I(t) represents the amplitude of the synaptic current, G_r_ is the resting conductance, and V_rest_ represents the resting membrane potential. These values were derived from the baseline current prior to each sound stimulation. V_m_ is the holding voltage, and G_e_ and G_i_ are the excitatory and inhibitory conductances, respectively. E_e_ (0 mV) and E_i_ (−70 mV) are the reversal potentials for excitation and inhibition, respectively. In this study, V_m_(t) was corrected according to V_m_(t) = V_hold_ − R_s_ × I(t), where V_hold_ was the command voltage, and R_s_ was the series resistance. A junction potential of approximately 10 mV was corrected. Then, G_e_ and G_i_ were calculated from this equation by holding the cell membrane at E_e_ and E_i_, respectively. G_e_ and G_i_ represent the pure excitatory and inhibitory synaptic input strengths, respectively.

Synaptic conductance was derived based on the assumption that the neurons were linear and isopotential, which can underestimate the absolute synaptic conductance amplitude. We minimized these absolute errors by comparing the conductance within a single cell. We used an integration model to simulate the membrane potential responses based on the synaptic conductance as follows:


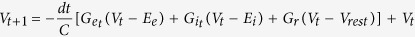


The values of G_r_ (15 nS) and C (100–150 pF) were based on experimental measurements. The resting membrane potential (V_rest_) was set to −70 mV.

### Statistics

Student’s t-tests or the paired t-tests were used for the comparisons between the groups with statistically equal variances. The F-test was used to evaluate the variance equivalence. The Shapiro–Wilk test was used to test the normality of the data.

## Additional Information

**How to cite this article**: Tao, C. *et al*. Bidirectional Shifting Effects of the Sound Intensity on the Best Frequency in the Rat Auditory Cortex. *Sci. Rep.* **7**, 44493; doi: 10.1038/srep44493 (2017).

**Publisher's note:** Springer Nature remains neutral with regard to jurisdictional claims in published maps and institutional affiliations.
